# Establishment and Application of Prostate Cancer Circulating Tumor Cells in the Era of Precision Medicine

**DOI:** 10.1155/2017/7206307

**Published:** 2017-11-05

**Authors:** Yoon Seok Suh, Jae Young Joung, Sung Han Kim, Ho Kyung Seo, Jinsoo Chung, Kang Hyun Lee

**Affiliations:** Center for Prostate Cancer, Hospital, National Cancer Center, Goyang, Gyeonggi-do, Republic of Korea

## Abstract

Prostate cancer (PC) is the second most common cancer in men and is the fifth leading cause of cancer-related deaths worldwide. Additionally, there is concern for overdiagnosis and overtreatment of PC. Thus, selection of an appropriate candidate for active surveillance as well as more accurate and less invasive tools for monitoring advanced PC is required. Circulating tumor cells (CTCs) have emerged as a liquid biopsy tool; there have been several reports on its role, technologies, and applications to various cancers, including PC. Liquid biopsy using CTCs has been gaining attention as a minimal invasive tool for investigation of biomarkers and for prognosis and assessment of response to therapies in patients with PC. Because of the lower invasiveness of liquid biopsy using CTCs, it can be performed more frequently; accordingly, personalized disease status can be successively determined at serial time points. CTC analysis enables detection of genomic alterations, which is drug-targetable, and it is a potential tool for monitoring response to therapeutic agents in patients with PC. This review focuses on the characteristics, technologies for analysis, and advantages and disadvantages of CTCs as a liquid biopsy tool and their application in PC. Finally, we propose future directions of CTCs.

## 1. Introduction

Prostate cancer (PC) is the second most common cancer in men and is the fifth leading cause of cancer-related deaths worldwide [1]. In the United States, PC is the second leading cause of cancer-specific deaths in men, and approximately 26,120 PC-related deaths were recorded in 2016 [2]. Although PC has a high prevalence among cancers as stated above, there is also a concern about overdiagnosis and overtreatment of PC. With an emerging concept of indolent PC [3], active surveillance, prescribed in several guidelines for active surveillance eligibility, of localized PC with low malignant potential is gaining wide acceptance [4–17]. However, after using the current method of prostate biopsy for diagnosing PC, there is a possibility of significant upgrade of PC [18]. Additionally, the patient undergoing active surveillance needs to undergo repeated prostate biopsies to determine disease progression. However, prostate biopsy by transrectal or transperineal approach is an invasive procedure and occasionally associated with morbidity, such as hematuria, hematochezia, acute urinary retention, urinary tract infection, and bacteremia [19]. Thus, to select an appropriate candidate for active surveillance, more accurate and less invasive tools for monitoring PC are required.

On the contrary, PC at an advanced stage is a lethal disease; however, several treatment strategies are available for locally advanced or metastatic PC, and overall survival (OS) in patients with even metastatic castration-resistant PC (CRPC) has been improved with the development of several promising drugs [20]. Quantitation of therapeutic response in metastatic CRPC is difficult due to lack of tools [21]. In a recent meta-analysis [22], circulating tumor cells (CTCs) positively indicated poor prognosis and CTC counts were a potential independent prognostic factor of survival rate in patients with CRPC. Hence, early detection of drug response to specific therapeutics and recurrence using CTCs would result in enhancing treatment outcome and reducing socioeconomic burden in treating PC.

Liquid biopsy using CTCs is gaining attention as a minimal invasive tool for investigation of biomarkers. Because of less morbidity of liquid biopsy using CTCs, it can be performed more frequently; accordingly, personalized disease status can be successively achieved at serial time points [23]. This is advantageous during treatment period for measuring tumor burden and early detection of recurrence or resistance [24]. Changes in CTC counts during systemic therapy can be used as a tool for monitoring treatment response [25].

Liquid biopsy using CTCs can aid in selection of an appropriate candidate for active surveillance and monitoring response to active surveillance or therapies and recurrence. Investigation of CTCs to discover new, more effective, and less invasive biomarkers is expected to contribute to reducing morbidity and costs associated with PC, the prevalence of which is increasing. In this review, we discussed the technology, advantages and disadvantages, applications, and the future direction of CTCs in PC.

## 2. Circulating Tumor Cells

Historically, CTCs were observed for the first time by Thomas Ashworth in the blood of a cadaver with metastatic cancer [26]. CTCs in the peripheral blood are originated from the primary tumor or metastatic foci and are circulated along the blood vessels and spread throughout the body [27]. They are involved in cancer metastasis, and protein expression and localization of CTCs at the cellular level were highly heterogeneous, which reflects the primary tumor and metastatic site [28]. CTCs have been detected in several malignancies, such as those of the prostate, breast, colon, lung, kidney, and bladder [29–37].

In the urological field, especially, elevation of CTC level in the peripheral blood was noted in patients with advanced renal cell carcinoma (RCC) and was associated with an aggressive phenotype [38]. CTC level in the peripheral blood was correlated with lymph node involvement and metastasis. CTC enumeration in the peripheral blood and expression of vimentin in CTCs were correlated with RCC progression [33]. CTCs were also detected in patients with metastatic bladder cancer [34, 35]. CTC levels were higher in the peripheral blood of patients with bladder cancer than in the control group. Quantification of CTCs using the expression of folate receptor *α* showed 82% sensitivity and 62% specificity in case of bladder cancer detection [36]. Enumeration of CTCs in the peripheral blood was an early predictor of bladder cancer recurrence and overall cancer-specific mortality [37].

There are several types of CTCs. Traditional CTCs were confirmed with a viability test, nuclear localization of cytokeratins, and an absence of CD45, which implies epithelial and nonhematopoietic origins, respectively [39]. They are larger cells with a subcellular feature or irregular morphology. Cytokeratin negative (CK−) CTCs or cancer stem cells undergo epithelial-mesenchymal transition (EMT) [40]. CK− CTCs are considered to have genomic profile or gene expression specific to cancer and morphology similar to a cancer cell and are prone to resistance and metastasis. Nuclear fragmentation or cytoplasmic bleb associated with apoptosis of CTCs was identified with Epic Sciences technology [40]. Efficacy of therapy can be checked by serial measurement of the ratio of traditional CTCs to apoptotic CTCs. Small CTCs are CD45− and CK+, and their morphology is similar to that of white blood cells [41]. CTC cluster is a bounded form of two or more CTCs [42, 43]. The CTC cluster is thought to contain CK− or small CTCs. Aceto et al. reported that this cluster is related to increased risk of metastasis and poor prognosis [42].

Small CTCs are related to differentiation into small cell carcinomas and progressive disease, which need a different treatment strategy.

Despite therapeutic and prognostic role of CTCs, detecting CTCs is difficult because they are present in small numbers [44]. The detection frequency of CTCs is 1–10 CTCs/mL of whole blood from patients with metastatic cancer [45]. Thus, fine and precise technologies with high sensitivity and specificity are required to detect various CTC subtypes in patients with numerous cancer types [40, 46].

## 3. Technologies Based on CTC Analysis

Analysis of CTCs includes their isolation and enrichment, detection, enumeration, and molecular characterization [47]. To isolate and enrich CTCs using filtration devices, physical features, such as size, electrical charge, and density, are considered [48].

The CellSearch® system (Veridex, Raritan, NJ, USA) was approved by the Food and Drug Administration (FDA) for monitoring patients with metastatic PC [49]. Currently, this is the only test for detecting CTCs that has received approval from the FDA. Briefly, 7.5 mL peripheral blood is sampled in an EDTA tube, centrifuged, and placed in the preparation system that enriches the tumor cells immunomagnetically with magnet and ferrofluid nanoparticles. CTCs of epithelial origin can be counted with a nuclear stain and a fluorescent antibody conjugate against EpCAM+; CD45−; and cytokeratins 8, 18+, and/or 19+ in the peripheral blood [43, 50]. For becoming CTCs, cells should possess a nucleus with diameter larger than 5 *µ*m and should be negative for the CD45 marker and positive for CK. If five or more CTCs are found, the result is considered positive. This test has a detection limit of 1 CTC/7.5 mL whole blood with 93% recovery capacity.

Although it is the only method approved by the FDA, there are several limitations. Specific equipment—the CellTracks AutoPrep and the CellTracks Analyzer II unit (Veridex LLC, Raritan, NJ, USA)—is required to perform the test. Considering the sensitivity and specificity, CTCs are not yet utilized practically; therefore, analyzing content in CTC, such as that of miRNAs, to detect cancer biomarkers is gaining interest [51]. Using antibodies that can recognize tumor marker may be biased due to requirement for enough expression of protein on the cell surface [52]. Some tumors do not express EpCAM and CKs, which can be downregulated during EMT [52].

Maintrac method employs microscopy for identifying CTCs [53]. For analysis, cells are prepared with single centrifugation and erythrocyte lysis. The processes of purifying or enriching cells can be omitted with this method. Instead, cells are identified among the mixture of blood components. EpCAM antibody is used for identification of cells. With this method, live EpCAM+ propidium-excluding cells can be counted as cancer cells. The suspension is analyzed by fluorescence microscopy, and the events are automatically counted. Previous studies demonstrated that adding CK- or CD45- specific antibodies does not have any merit [54, 55]. Maintrac uses the dynamics of cell count, and varying tumor cell number is an indicator of cancer activity. This method was used for determining the outcome of chemotherapy and for monitoring the response to hormone therapy [54, 56–58]. Additionally, early detection of cancer recurrence was verified with this method [59, 60].

However EpCAM+ cells can be detected in the peripheral blood when inflammation disease or skin burns are present [61]. Thus, diagnosis of cancer by EpCAM+ cells is not appropriate.

CTCs can be separated based on antigen-antibody interactions. Antibodies against tumor specific biomarkers, such as PSA, Her2, and EpCAM, are used. Magnetic-activated cell sorting (MACS) is a commonly used separation method based on magnetic nanoparticles. Microfluidic separation and immunomagnetic assay were used in previous studies [62–66]. Viruses with oncolytic features were used for detecting CTCs [67]. In other studies, for better control of the magnetic field, magnetic structure with a microscale was implemented [68–70].

A filtering-based method considering cell size was employed to capture CTCs [71]. The ScreenCell method captures CTCs with an isolation device using the drawn peripheral blood for 4 hours [72]. It allows isolation of CTCs by a filtration-based device using the whole blood.

Previous studies reported detection of CTCs by GILUPI GmbH* in vivo* [73, 74]. By inserting a metal wire that is coated with an antibody into a peripheral vein for 30 minutes, CTCs bind to the antibodies. After isolating CTCs, several methods, including immunofluorescence staining and molecular genetics, could be employed [67, 75]. Analyzing higher blood volume for detecting CTCs is an advantage of this method. Viatar CTC Solutions developed a method based on therapeutic oncopheresis that uses a mechanical filtration system for dialysis of CTCs for 4 hours [76].

CTC detection can be performed using RNA- or DNA-based technology with improved sensitivity. AdnaTest kit (Qiagen, Hannover, GE) uses simultaneous amplification and detection of multiple transcripts of circulating RNA or DNA to detect CTCs [77]. This method utilizes multiplex reverse transcription polymerase chain reaction (RT-PCR). In addition, other methods, such as CTC filters, acoustic-based separation, and microscopy, could be used to detect CTCs [78–80].

Methods for CTC isolation should allow their identification, enumeration, and characterization. ViewRNA ISH Cell Assays is a method that enables multiplex, single-molecule detection of specific RNA target with in situ hybridization technology [81]. Proprietary probe design allows high sensitivity and specificity using background suppression and branched DNA signal amplification.

After removal of the primary tumor, biopsy of the tumor by tissue typing is not possible [82]. In this case, tissue sections from the primary tumor can be used for typing, and CTC characterization can be performed to identify the tumor phenotypes. FISH assays were used on CTCs and identification of Her2, IGF-1R, Bcl-2, AR status, ERG, and PTEN was performed [83–86].

## 4. Advantages and Disadvantages of CTCs as a Liquid Biopsy Tool

Tissue biopsy is invasive, unsuitable to be performed repeatedly, and unpredictive for understanding effectiveness of treatment, disease progression, and metastasis risk [87]. CTCs can render ongoing information of metastasis reflecting the patient's disease status [83]. CTC detecting method using the peripheral blood is feasible and safe to be performed. Additionally, successive and repeated sampling is available. These characteristics of liquid biopsy allow monitoring the disease status, including progression and response to therapies.

Despite the aforementioned merits of methods for CTC detection, some demerits should be overcome before adopting it practically; several clinical, technical, and biological problems need to be solved. Because tumor is characteristically heterogeneous, revealing the source of CTCs is the most important to understand its clinical usefulness.

Since CTCs are present at low levels in the whole blood, assays with higher sensitivity are required. Droplet digital PCR (ddPCR) is a promising method, but sufficient blood is needed for appropriate analysis. Another hurdle is measuring the changes in CTC counts that might be minute. These demerits should be addressed to consider CTCs as a monitoring marker for cancer.

Since CTCs are extremely rare in the blood, sensitivity and specificity are not enough for accurate detection of CTCs, and enumeration has not been accepted as a method for tumor staging, CTCs cannot be used routinely in clinical practice yet. Despite the presence of various methods for CTC detection, none of them are established to be clinically applicable due to narrow detection spectrum, low purity, and loss of CTCs [116]. Additionally, methods for CTC detection are usually accompanied by complicated processes, long time for CTC detection, and significant costs.

## 5. Application of CTCs in PC

Studies that reported CTC detection in patients with PC are summarized in Table 1. Some studies demonstrated the association of CTCs with biochemical recurrence after radical prostatectomy. In the study with 250 high-risk patients with PC, presence of prostate stem cell antigen (PSCA) mRNA in the peripheral blood was reported to be a significant predictor of biochemical recurrence after radical prostatectomy (HR, 4.549; 95% CI, 1.685–12.279) [93]. Joung et al. reported that prostate specific membrane antigen (PSMA) mRNA in the peripheral blood was a predictor of biochemical recurrence after radical prostatectomy [95]. Nested RT-PCR assay detecting PSMA mRNA-bearing cells in the peripheral blood was employed to detect CTCs. PSMA mRNA (HR, 3.697; 95% CI, 1.285–10.634;* P* = 0.015) was an independent predictor of biochemical recurrence.

CTCs are often found in patients with mCRPC and it showed a prognostic significance [102]. de Bono et al. reported that elevated level of CTCs was a prognostic factor in patients with mCRPC [89]. Unfavorable group that had 5 or >5 CTCs/7.5 mL peripheral blood showed significantly shorter OS (median OS 11.5 versus 21.5 months) than that showed by favorable group (≤5 CTCs/7.5 mL peripheral blood). In this study, level of CTCs was a stronger predictor of OS than PSA was.

In a phase III clinical study, CTC level elevation after three cycles of docetaxel with lenalidomide chemotherapy in patients with mCRPC predicted poor survival [114]. Scher et al. reported in their phase III trial that level of lactate dehydrogenase and number of CTCs in the whole blood were predictors of OS in patients with mCRPC that had been treated with abiraterone acetate and docetaxel [105]. Two-year survival of patients with ≥5 CTCs/7.5 mL was 2%, while that of patients with <5 CTCs/7.5 mL was 46% (CTCs were counted at 12 weeks). Lorente et al. demonstrated that decline in CTC count by 30% after treatment from an initial count ≥5 cells/7.5 mL is independently associated with CRPC OS following chemotherapy and abiraterone treatment [113].

CTCs have emerged as a biomarker in mCRPC that guides therapeutic decisions. For evaluating predictor of resistance to treatments, molecular alterations in androgen receptor (AR) on CTCs were investigated. Antonarakis et al. demonstrated an association of detection of AR splice variant-7 (AR-V7) in CTCs and resistance to AR-targeting treatments in patients with mCRPC [103]. Recently, a real-time CTC-based assay of nuclear AR expression in CTCs of patients with CRPC was developed using CellSearch System [109].

Recently, no significant correlation between detection of AR-V7 positive CTCs and primary resistance to taxane chemotherapy was demonstrated in patients with mCRPC [111, 117]. Thalgott et al. reported that categorical status of CTC count, which was assessed after a cycle of taxane chemotherapy, was a predictor for progression-free survival and OS in patients with mCRPC [107]. This implies that categorical CTC count can act as a predictor of treatment response to taxane chemotherapy. However, AR-V7 status was not a predictor of response to cabazitaxel [110, 118].

## 6. Future Directions of CTCs

Cancer biomarker development using CTCs is a promising and rapidly expanding field. The emergence of ddPCR and next-generation sequencing (NGS) enabled improved detection rate and minimized time and expenses. Gulati et al. reported biomarker panel with an improved sensitivity and specificity compared to those of previous single markers [119]. However, most of the other markers are still experimental; accordingly establishment of these markers is required in the future. For improving accuracy of CTCs as a biomarker for cancer activity, usage of different molecular alteration levels, such as combining proteomics, genomics, and transcriptomics, would be beneficial [120, 121].

The analysis technique of CTCs has to be innovated with high selectivity and sensitivity that can be utilized for CTC purification, downstream CTC characterization, and retaining viable CTCs for ex vivo expansion [122]. Various CTC detection methods may count different subclasses of CTCs, and the best CTCs, which are clinically relevant, have not been established. Thus, the best analysis methods should be investigated for use as a biomarker of cancer and monitoring response to therapies.

## 7. Conclusion

In the era of precision medicine, treatment of PC will be tailored based on behavior of PC in each patient. Liquid biopsy using CTCs is a promising tool as a marker for prognosis and assessment of response to therapies in patients with PC. CTC analysis has a minimal invasive nature; accordingly, it is a suitable follow-up of biopsy, when serial PC behavior is required. CTC analysis enables detection of genomic alterations, which is drug-targetable, and it is a potential tool for monitoring response to therapeutic agents in patients with PC.

The utility of CTCs in patients with CRPC and localized PC after radical prostatectomy has been reported. Identification of gene expression, such as AR expression variants in CTCs, can be used as promising marker for selection of therapeutics in patients with mCRPC. However, application of CTCs in candidate for active surveillance is not well evaluated; accordingly, development of CTC marker that is specific in patients eligible to current active surveillance criteria is required for reflecting the increasing trend of active surveillance.

Future research on CTCs should be focused on developing a marker for CTCs with improved sensitivity and specificity, prior to their application to the detection of PC. The ideal marker for PC should be expressed on most of the CTCs, but not on other cells in the blood, and its expression should be maintained throughout the course of PC. The effort for using different molecular alteration levels has to be made for improving accuracy of CTCs as well. Finally, the technique used for CTC analysis should be improved to obtain high selectivity and sensitivity for the application of CTCs in PC.

## Figures and Tables

**Table 1:** Studies that reported detection of circulating tumor cells in patients with prostate cancer.

Study	Number of patients	CTCs enrichment method	Markers	Stage of disease	Treatment
Danila et al. [88] (2007)	120	CellSearch	CD45− CK+	CRPC	Chemotherapy
de Bono et al. [89] (2008)	231	CellSearch	CD45− CK+	CRPC	Chemotherapy
Goodman Jr. et al. [90] (2009)	100	CellSearch	CD45− CK+	CRPC	Chemotherapy
Scher et al. [91] (2009)	156	CellSearch	CD45− CK+	CRPC	Surgery/chemotherapy
Olmos et al. [92] (2009)	119	CellSearch	CD45− CK+	CRPC	Chemotherapy
Joung et al. [93] (2010)	103	Nested RT-PCR	PSCA-mRNA+	High-risk PC	Radical prostatectomy
Coumans et al. [94] (2010)	179	CellSearch	EpCAM+ CD45− CK+	CRPC	Chemotherapy
Joung et al. [95] (2010)	134	Nested RT-PCR	PSMA mRNA+	Localized PC	Radical prostatectomy
Strijobs et al. [96] (2010)	154	CellSearch	CD45− CK+	CRPC	Docetaxel based chemotherapy
Danila et al. [97] (2011)	48	CellSearch	CD45− CK+	CRPC	Abiraterone
Scher et al. [98] (2013)	144	CellSearch	CD45− CK+	CRPC	Cabozantinib
Thalgott et al. [99] (2013)	55	CellSearch	CD45− CK+	CRPC	Docetaxel based chemotherapy
Okegawa et al. [100] (2014)	57	CellSearch	CD45− CK+	CRPC	Docetaxel based chemotherapy
Danila et al. [101] (2014)	97	RT-PCR assay	Not reported	CRPC	Not reported
Goldkorn et al. [102] (2014)	263	CellSearch	CD45− CK+	CRPC	Docetaxel based chemotherapy
Antonarakis et al. [103] (2014)	62	AdnaTest	PSA+ PSMA+or EGFR+	CRPC	Enzalutamide and abiraterone
Chang et al. [104] (2015)	70	CellSearch	Not reported	CRPC	Docetaxel based chemotherapy
Scher et al. [105] (2015)	711	CellSearch	CD45− CK+	CRPC	Abiraterone or prednisone
Fleisher et al. [106] (2015)	258	CellSearch	EpCAM+ CD45− CK+	CRPC	Enzalutamide
Thalgott et al. [107] (2015)	33	CellSearch	EpCAM+ CK+ nucleic acid+	CRPC	Docetaxel based chemotherapy
Lorente et al. [108] (2015)	439	CellSearch	CD45− CK+	CRPC	Abiraterone or chemotherapy
Crespo et al. [109] (2015)	48	CellSearch	CD45− CK+	CRPC	Enzalutamide and abiraterone
Onstenk et al. [110] (2015)	29	CellSearch	CD45− CK+	CRPC	Cabazitaxel based chemotherapy
Antonarakis et al. [111] (2015)	37	AdnaTest	PSA+ PSMA+ or EGFR+	CRPC	Chemotherapy
Bitting et al. [112] (2015)	89	CellSearch	CD45− CK+	CRPC	Chemotherapy
Lorente et al. [113] (2016)	486	CellSearch	CD45− CK+	CRPC	Abiraterone plus prednisone or prednisone
Vogelzang et al. [114] (2017)	208	CellSearch	CD45− CK+	CRPC	Docetaxel based chemotherapy
Tsumura et al. [115] (2017)	59	CellSearch	DAPI+ CK+ CD45−	Nonmetastatic PC	Brachytherapy

CTC: circulating tumor cell; CK: cytokeratins; CRPC: castrate-resistant prostate cancer; EpCAM: epithelial cell adhesion molecule; RT-PCR: real-time polymerase chain reaction; PSCA: prostate stem cell antigen; PC: prostate cancer; PSMA: prostate specific membrane antigen; EGFR: epidermal growth factor receptor.
